# EStradiol and PRogesterone in In vitro ferTilization (ESPRIT): a multicenter study evaluating third- versus second-generation estradiol and progesterone immunoassays

**DOI:** 10.1007/s40618-020-01211-x

**Published:** 2020-03-13

**Authors:** N. P. Polyzos, E. Anckaert, P. Drakopoulos, H. Tournaye, J. Schiettecatte, H. Donner, G. Bobba, G. Miles, W. D. J. Verhagen-Kamerbeek, E. Bosch

**Affiliations:** 1 Department of Reproductive Medicine, Dexeus University Hospital, Gran Via Carles, 11171-75, 08028 Barcelona, Spain; 2 Faculty of Medicine and Pharmacy, Vrije Universiteit Brussel, Laarbeeklaan 101, 1090 Brussels, Belgium; 3 Department of Clinical Medicine, Faculty of Health, Aarhus University Incuba/Skejby, Building 2, Palle Juul-Jensens Boulevard 82, Aarhus N, 8200 Aarhus, Denmark; 4 Laboratory of Hormonology and Tumour Markers, Universitair Ziekenhuis Brussel, Free University of Brussels, Brussels, Belgium; 5 Centre for Reproductive Medicine, University Hospital (UZB), Laarbeeklaan 101, 1090 Jette, Brussels Belgium; 6 Medical and Scientific Affairs, Roche Diagnostics GmbH, Nonnenwald 2, 82377 Penzberg, Germany; 7 Medical and Scientific Affairs, Roche Diagnostics International Ltd, Forrenstrasse 2, 6343 Rotkreuz, Switzerland; 8 Biostatistics and Data Management, Roche Diagnostics Operations Inc., 9115 Hague Road, Building R, Indianapolis, IN 46256 USA; 9 Human Reproduction Unit, IVI-RMA, Plaza de la Policia Local 3, 46015 Valencia, Spain

**Keywords:** Estradiol, Progesterone, Immunoassay, Ovarian stimulation, In vitro fertilization

## Abstract

**Purpose:**

To assess estradiol (E_2_) and progesterone levels during ovarian stimulation determined by third-generation (Gen III) and second-generation (Gen II) Elecsys^®^ immunoassays.

**Methods:**

E_2_ and progesterone concentrations were measured using Elecsys^®^ Gen III and Gen II immunoassays, and progesterone concentrations on the day of ovulation triggering were determined by LC–MS/MS. This was a retrospective, non-interventional study conducted at European tertiary referral infertility clinics in women aged 18–45 years, with a body mass index 18–35 kg/m^2^, regular menses, and both ovaries.

**Results:**

Serum samples were obtained from 230 women classified by oocyte retrieval as poor (33.0%; 0–3 oocytes), normal (40.9%; 4–15 oocytes), or high (26.1%; > 15 oocytes) responders. E_2_ and progesterone levels increased during ovarian stimulation, with greatest increases observed in high responders. Elecsys^®^ Gen III and Gen II assay results were highly correlated for E_2_ (Pearson’s *r* = 0.99) and progesterone (*r* = 0.89); Gen III results were lower than Gen II for both E_2_ and progesterone. On the day of triggering, Gen III E_2_ and progesterone levels showed a difference of − 15.0% and − 27.9%, respectively. Progesterone levels (on day of triggering) measured by LC–MS/MS correlated better with Gen III (0.98) than Gen II (0.90). Mean relative differences for Gen III and Gen II assays versus LC–MS/MS were 14.6% and 62.8%, respectively.

**Conclusion:**

E_2_ and progesterone levels determined with Elecsys^®^ Gen II and III assays were highly correlated; results were lower for Gen III versus Gen II. Differences observed for progesterone on the day of triggering may be clinically relevant.

**Electronic supplementary material:**

The online version of this article (10.1007/s40618-020-01211-x) contains supplementary material, which is available to authorized users.

## Introduction

The measurement of estradiol (E_2_) and progesterone is an essential part of ovarian stimulation for in vitro fertilization (IVF) and intracytoplasmic sperm injection (ICSI). The accurate monitoring of E_2_ levels, along with ultrasound, are key aspects of IVF that support dose adjustment and evaluation of ovarian hyperstimulation syndrome risk and potential cycle cancellation. Serum progesterone measurement during the follicular phase has become part of routine clinical practice in the last decade, mainly due to the inverse association between late follicular phase progesterone levels and pregnancy rates [1, 2].

As hormone measurement during IVF or ICSI cycles is important for optimizing ovarian response and treatment safety and efficacy, several different assays have been developed for the measurement of E_2_ and progesterone serum levels. Automated analyzers and direct immunoassays are commonly used, but previous studies have shown that different assays demonstrate various degrees of bias and interlaboratory variability [1, 3–7]. Liquid chromatography (LC) coupled with mass spectrometry (MS) methods have the potential to provide greater specificity and sensitivity than immunologic methods, but doubts have been expressed over discrepancies between different assays [8]. Moreover, the “gold standard” method of isotope dilution (ID) with gas chromatography (GC)/MS (ID-GC/MS) is complex, and the throughput is too low for routine clinical use [8]. Modern assays need to fulfil a number of criteria: have good precision to characterize patient response to treatment; be reliable over a wide concentration range; have high specificity for E_2_ and be able to exclude exogenous estrogens; and provide good interlaboratory reproducibility [8]. The Elecsys^®^ second-generation (Gen II) Estradiol and Progesterone immunoassays have been widely used for monitoring serum E_2_ and progesterone levels. The Elecsys^®^ third-generation (Gen III) Estradiol and Progesterone immunoassays have demonstrated good correlation with Gen II assays during assay development, but IVF patient samples were not available for validation.

Given the lack of evidence, the ESPRIT (EStradiol and PRogesterone in In vitro ferTilization) study was conducted to determine E_2_ and progesterone levels during ovarian stimulation in a population of poor, normal, and high responders [9] who were undergoing IVF using gonadotropin-releasing hormone (GnRH) agonist or antagonist protocols in a routine clinical setting. The ESPRIT study aimed to compare the recently developed Elecsys^®^ Estradiol and Progesterone Gen III assays with the respective commonly used Gen II assays. The Elecsys^®^ Gen III and Gen II Progesterone assays were also compared with a LC–MS/MS method to further assess assay performance.

## Materials and methods

### Study design

ESPRIT was an exploratory, retrospective, non-interventional, multicenter study. The study used serum samples that were originally collected during the stimulation cycle from patients on GnRH agonist or antagonist protocols at two tertiary referral infertility clinics in Europe (IVI-RMA Valencia [IVI], Spain; UZ Brussel [UZB], Belgium).

The study included stored samples from women aged 18–45 years, with a body mass index of 18–35 kg/m^2^, and who had both ovaries present and regular ovulatory menses (every 25–35 days). Samples were excluded if women had current or past disease affecting the ovaries or gonadotropin/sex steroid hormone levels, polycystic ovary syndrome, or any known untreated endocrine abnormality, or were undergoing hormone therapy at the time of blood sampling.

### Ethics approval

All participants provided written informed consent prior to enrolment. The protocol was approved by the Clinical Research Ethics Committee of the IVI-RMA Valencia (December 20, 2016) and the Medical Ethics Committee of the Free University of Brussels (January 25, 2017).

### Immunoassays

The Elecsys^®^ Estradiol Gen III and Gen II assays are used for in vitro quantitative determination of E_2_ in human serum and plasma on **cobas e** analyzers (Roche Diagnostics GmbH, Mannheim, Germany). Measuring ranges for each assay (defined by the lower limit of detection and the maximum of the master curve) are as follows: Elecsys^®^ Estradiol Gen III assay, 18.4–11,010 pmol/L (5.0–3000 pg/mL); Elecsys^®^ Estradiol Gen II assay, 18.4–15,781 pmol/L (5.0–4300 pg/mL) (Roche, data on file). Sample dilution allows measurement of higher levels up to 30,000 pg/mL for Gen III (recommended dilution ratio of 1:10) and up to 21,500 pg/mL for Gen II (recommended dilution ratio of 1:5). Standardization is achieved in the E_2_ assays using a panel of samples with ID-GC/MS-assigned target values [10]. The Gen II E_2_ assay uses polyclonal antibodies, whereas the Gen III E_2_ assay uses high-affinity monoclonal antibodies, which permit higher sensitivity. The Gen III assay also uses a lower concentration of antibodies than the Gen II assay, and thus, cross-reactivity is lower for Gen III. Clinical and Laboratory Standards Institute (CLSI) protocol EP05-A2 precision and repeatability profiles for the Gen III assay are similar to the Gen II assay on the **cobas e** 601 analyzer, and are improved at some concentrations (Roche, data on file).

The Elecsys^®^ Progesterone Gen III and Gen II assays determine progesterone levels in human serum and plasma on **cobas e** analyzers. Measuring ranges for each assay are as follows: Elecsys^®^ Progesterone Gen III, 0.159–191 nmol/L (0.050–60.0 ng/mL); Elecsys^®^ Progesterone Gen II, 0.095–191 nmol/L (0.030–60.0 ng/mL) (Roche, data on file). The Elecsys^®^ Progesterone Gen II assay employs mouse monoclonal antibodies, whereas the Elecsys^®^ Progesterone Gen III assay uses sheep monoclonal antibodies, which provide higher specificity for progesterone (Roche, data on file) and reduced cross-reactivity against major steroids and metabolites.

### LC–MS/MS

Progesterone concentrations determined by the Elecsys^®^ Progesterone Gen II and Gen III assays were compared with those measured by LC–MS/MS in 148 samples at UZB (treatment groups: GnRH agonist, *n* = 60; GnRH antagonist, *n* = 88; response groups: poor, *n* = 58; normal, *n* = 60; high, *n* = 30). The determination of serum progesterone is based on ID–LC–MS/MS, subsequent to a sample preparation procedure involving protein precipitation and ultra-centrifugation. Dissolved deuterated progesterone was added as an internal standard and simultaneously as a protein precipitation reagent to samples and calibrators. After precipitation, the samples were centrifuged and the supernatant was transferred to centrifugation filters. The filtrate of unknown serum samples and calibrators was injected into the high-performance LC system to separate the analytes from other matrix components, using an analytical column in the reverse-phase mode. Each analyte was then detected in the MS (TSQ Quantum Ultra; Thermo Fisher Scientific) with atmospheric pressure ionization and in selected reaction monitoring mode. Quantification was based on the area ratio between analyte and internal standard. Further details are available in Online Resource 1.

### Study procedures

Demographic and health assessment information was collected for all patients. Samples were collected according to routine clinical practice at each site (IVI: Visit 1, Day 5 ± 1 of ovarian stimulation; Visit 2, Day 7 ± 1; Visit 3, Day 9 + 3; Visit 4, day of ovulation triggering; UZB: Visit 1, Day 0/1 of ovarian stimulation; Visit 2, Day 8; Visit 3, Day 10–11; Visit 4, day of ovulation triggering). All follicles ≥ 10 mm were measured in sagittal and transverse planes by 2D ultrasound, and the mean diameter of each follicle was recorded; small (< 12 mm), intermediate (12–15 mm), and mature (≥ 16 mm) follicle counts were recorded at each visit.

E_2_ and progesterone concentrations in each sample were determined retrospectively using the Elecsys^®^ Gen III and Gen II assays (per the manufacturer’s instructions) on **cobas e** 601 (UZB) or **cobas e** 411 (IVI) analyzers. Progesterone concentrations in the samples collected on the day of ovulation triggering were also determined by LC–MS/MS (UZB only).

### Statistical methods

A simulation was performed to estimate the precision of the 95% confidence interval (CI) for the difference between Elecsys^®^ Estradiol Gen III and Gen II assays (E_2_ has a greater measuring range and a larger bias between assay generations than progesterone). Based on these calculations and pooling across timepoints (*N* = 120), a sample size of ≥ 30 patients per subgroup would attain an average precision on the 95% CI of differences between assay generations of ± 2.7% of the mean E_2_ value. We assumed a bias between Gen III and Gen II assays, consistent with that reported from data on technical performance studies with other systems, which could be extended to the measurement range expected for this study: Passing–Bablok 0.86 (E_2_ Gen II) + 8.27; E_2_ range 1450–10,000 pmol/L [11]. The bias is constant over stimulation cycles and on day of ovulation triggering.

Patient baseline characteristics/demographics were summarized using descriptive statistics (mean, median, standard deviation [SD], minimum, maximum, and percentiles [5%, 25%, 75%, and 95%]). E_2_ and progesterone concentrations were presented by site, GnRH therapy protocol (agonist/antagonist), and ovarian response (poor/normal/high). To account for potential effects of different gonadotropin use, E_2_ levels were calculated per mature follicle on day of ovulation triggering. Responses to ovarian stimulation were predefined based on the number of oocytes retrieved following ovarian stimulation as poor (0–3), normal (4–15), or high (> 15). Mean relative difference (Gen III vs. Gen II) and associated SDs, range, and upper and lower limits (equal to ± 2SD) were calculated relative to Gen II for E_2_ and progesterone. Method comparisons (Gen III vs. Gen II; Progesterone Gen III vs. LC–MS/MS; Progesterone Gen II vs. LC–MS/MS) were performed using Passing–Bablok regression analysis and Pearson’s correlation coefficients were calculated; bias was examined using Bland–Altman analysis. Analyses were performed in subgroups at truncated ranges to assess variability at the proposed 1.5 ng/mL progesterone threshold (obtained previously using AxSYM system [Abbott Cientifica S.A., Madrid, Spain]) [1, 12, 13]. Statistical analyses were performed using SAS software (version 9.4; SAS System for Windows 7) and R (version 3.4.0). Regression analyses were performed using the R package mcr (version 1.2.1).

## Results

### Study population

Patient characteristics were representative of a European population (Online Resource 2). In the overall cohort of 230 women, median age was 36 years (range, 25–44 years) and 67.8% of women were White/Caucasian. Patients were classified as poor responders (0–3 oocytes; *n* = 76 [33.0%]), normal responders (4–15 oocytes; *n* = 94 [40.9%]), or high responders (> 15 oocytes; *n* = 60 [26.1%]) based on the number of oocytes retrieved; as expected, response to ovarian stimulation decreased with age (Online Resource 2).

A total of 62 patients received a GnRH agonist protocol and 168 patients received a GnRH antagonist protocol. Median age was comparable in the overall population and in subgroups of patients who underwent GnRH agonist or antagonist protocols.

### E_2_ and progesterone levels and response status

**Fig. 1:**
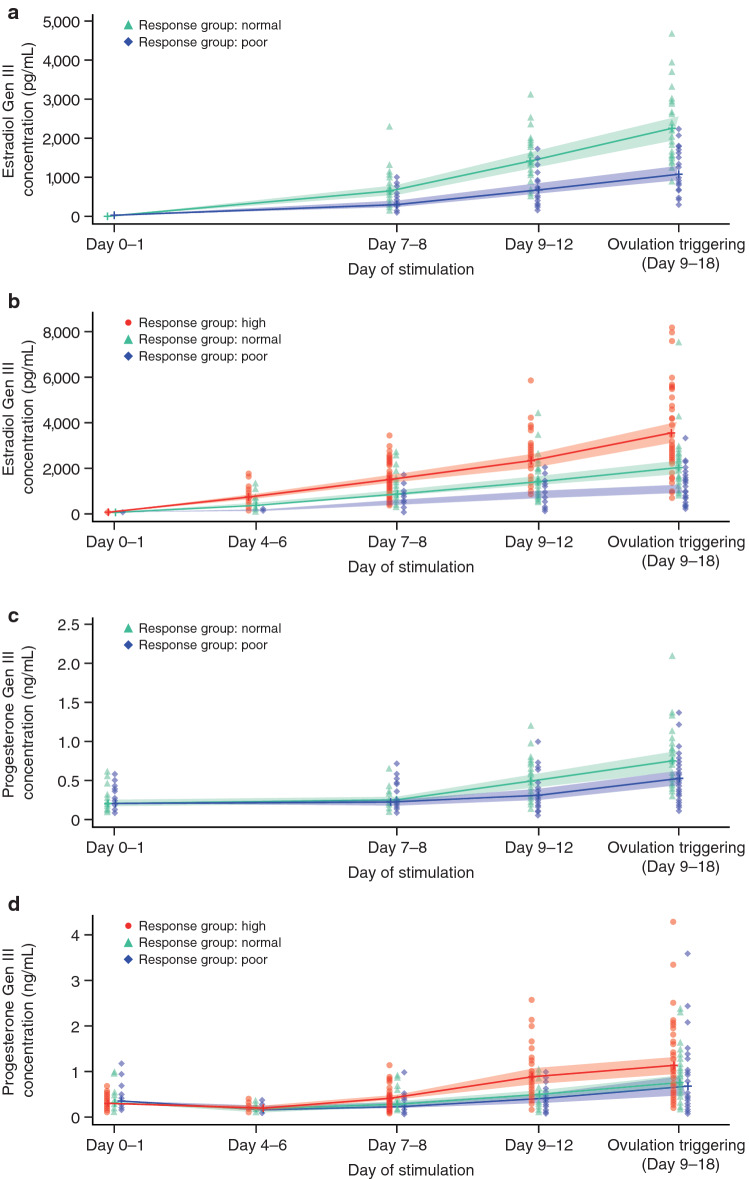
Change in estradiol concentration measured using the Elecsys^®^ Estradiol Gen III assay by ovarian response group (poor, 0–3 oocytes; normal, 4–15 oocytes; high, > 15 oocytes) following gonadotropin-releasing hormone **a** agonist and **b** antagonist treatment. Change in progesterone concentration measured using the Elecsys^®^ Progesterone Gen III assay by ovarian response group (poor, 0–3 oocytes; normal, 4–15 oocytes; high, > 15 oocytes) following gonadotropin-releasing hormone **c** agonist and **d** antagonist treatment

E_2_ and progesterone levels increased during ovarian stimulation, with greater increases in high responders versus poor or normal responders (Fig. 1). On the day of ovulation triggering, progesterone levels increased versus previous visits in both agonist- and antagonist-treated patients, regardless of responder status. The ratios of mean analyte concentration to mature follicle count on day of ovulation triggering were in the range 324–411 (E_2_ Gen III) and 0.11–0.24 (progesterone Gen III) across protocols and response groups, based on follicle counts (Online Resource 3 and 4).

### Method comparison of Gen III and Gen II assays

Method comparison based on Passing–Bablok regression demonstrated a good correlation between Elecsys^®^ Gen III and Gen II assays for E_2_ (*y* = 0.86x–4.35; 0–12,500 pg/mL; Pearson’s *r* = 0.99), but correlation was weaker for progesterone (*y* = 0.86x–0.20; 0–5 ng/mL; Pearson’s *r* = 0.89).

**Fig. 2:**
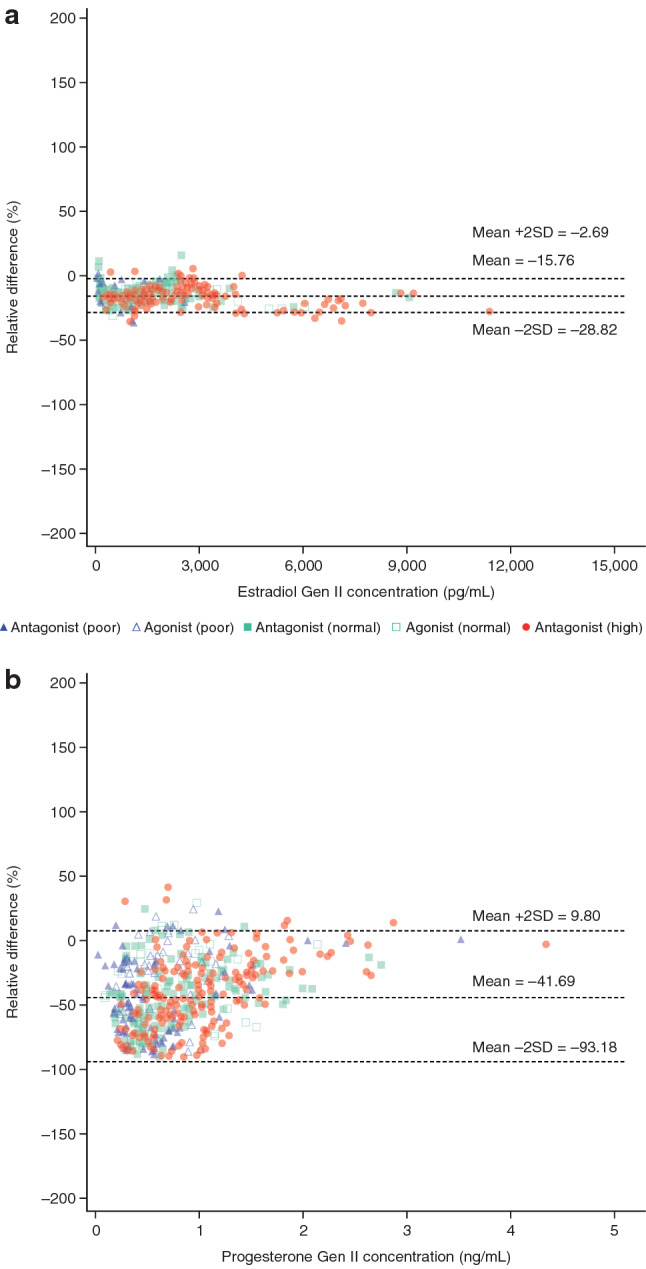
Bland–Altman plots for the comparison of **a** estradiol and **b** progesterone concentrations measured using the Elecsys^®^ Gen III and Gen II assays. *2SD* two standard deviations

E_2_ concentrations were lower when measured with the Gen III assay versus the Gen II assay (Figs. 1a–b, 2a; Table 1; Online Resource 5 and 6). The mean relative difference between Gen III and Gen II assays was − 15.8% (SD, 6.5%; *N* = 684 samples) across visits; on the day of ovulation triggering, the mean relative difference was − 15.0%. Bland–Altman analysis showed a constant bias up to ~ 3000 pg/mL; at concentrations > 3000 pg/mL, Gen III had a lower recovery than Gen II (Fig. 2a).

**Table 1:** Relative difference in E_2_ and progesterone levels as measured with the Elecsys^®^ Gen III and Gen II assays

Test	Measurement range	*N*	Relative difference (%)			
			Mean (SD)	Min, max	Lower limit (mean relative difference − 2SD)	Upper limit (mean relative difference + 2SD)
E_2_ (pg/mL)	Full	684	− 15.76 (6.53)	− 35.71, 14.29	− 28.82	− 2.69
Progesterone (ng/mL)	Full	674	− 41.69 (25.75)	− 90.91, 42.66	− 93.18	9.80
	Truncated: 0–1	493	− 46.32 (25.37)	− 90.91, 42.66	− 97.06	4.43
	Truncated: > 1–1.5	129	− 33.55 (22.16)	− 86.12, 25.00	− 77.87	10.78
	Truncated: > 1.5	52	− 18.04 (18.97)	− 67.32, 16.22	− 55.97	19.89

Data are combined by protocol, response group, and site (Days 0–1 excluded); differences are expressed as Elecsys^®^ Gen III versus Gen II assays

*2SD* two standard deviations, *E*_*2*_ estradiol, *SD* standard deviation

Progesterone concentrations were also lower when measured with the Gen III assay compared with the Gen II assay (Figs. 1c–d, 2b; Table 1; Online Resource 5 and 7). The mean relative difference between Gen III and Gen II assays was − 41.7% (SD, 25.8; *N* = 674 samples) across visits; on the day of ovulation triggering, the mean relative difference was − 27.9%. For 20/36 (56%) patients with Gen II progesterone levels > 1.5 ng/mL, results for Gen III were concordant; however, 16/36 (44%) patients had a progesterone level < 1.5 ng/mL with Gen III.

### Method comparison of progesterone Gen III and Gen II assays versus LC–MS/MS

**Fig. 3:**
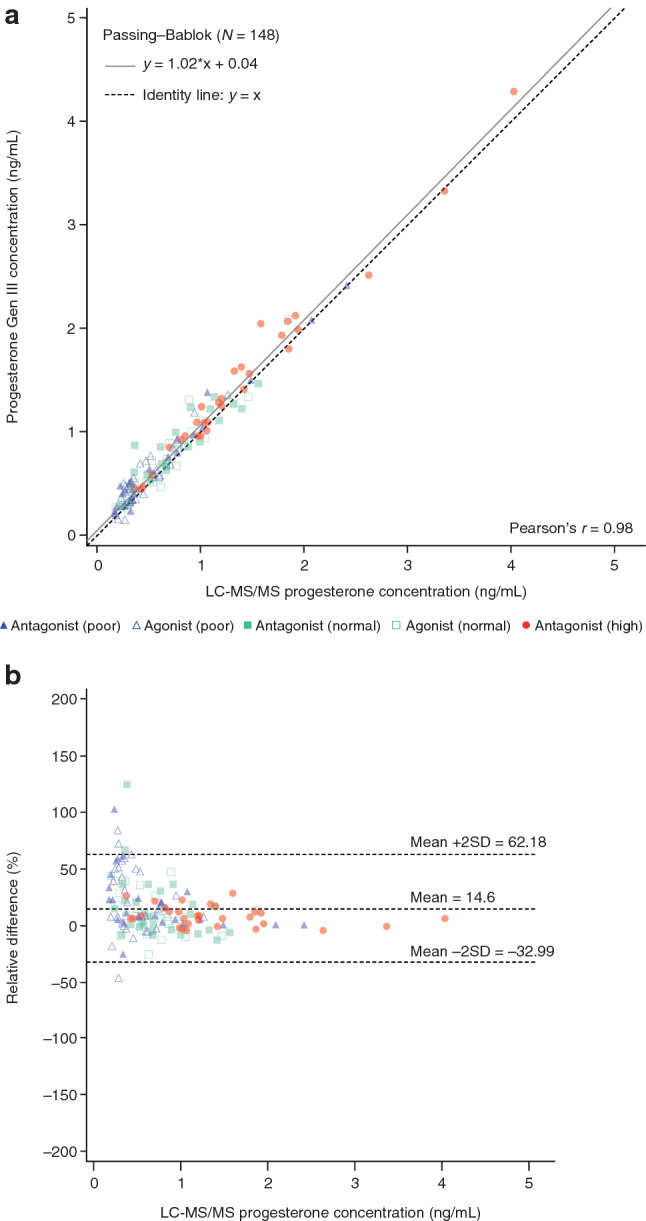
**a** Passing–Bablok regression analysis and **b** Bland–Altman plot for the comparison of progesterone concentrations measured using the Elecsys^®^ Progesterone Gen III assay and LC–MS/MS. *2SD* two standard deviations, *LC–MS/MS* liquid chromatography–tandem-mass spectrometry

Passing–Bablok analyses showed a higher correlation between the progesterone Gen III assay and LC–MS/MS (*y* = 1.02*x + 0.04; Pearson’s *r* = 0.98; *N* = 148 samples; Fig. 3a) compared with the progesterone Gen II assay and LC–MS/MS (*y* = 1.01*x + 0.28; Pearson’s *r* = 0.90; *N* = 148 samples; Online Resource 8).

Bland–Altman analyses showed that the mean (SD) relative difference versus LC–MS/MS was lower with Gen III (14.6% [23.8%]) than with Gen II (62.8% [69.2%]) (Fig. 3b; Online Resource 8 and 9); this trend was most pronounced for progesterone concentrations < 0.5 ng/mL. Relative to LC–MS/MS, a positive bias was observed with Gen II across the whole measuring range (0–5 ng/mL; Online Resource 8) compared with a more uniform bias (trending toward positive) with Gen III (Fig. 3b). There were fewer samples above the proposed 1.5 ng/mL threshold with Gen III (16/148 [10.8%]) compared with Gen II (28/148 [18.9%]) (Online Resource 9).

## Discussion

Measurement of hormone levels during ovarian stimulation has an important role in optimizing the likelihood that IVF will result in pregnancy. Accurate and reliable methods to measure E_2_ and progesterone are essential to assess treatment response, and support clinical decision-making relating to dose adjustment and the assessment of ovarian hyperstimulation syndrome risk. Our findings demonstrate that E_2_ levels determined with the Elecsys^®^ Estradiol III assay are highly correlated with results determined using the well-established Elecsys^®^ Estradiol II assay (*r* = 0.99); the correlation between Elecsys^®^ Progesterone III and Elecsys^®^ Progesterone II assay results was slightly weaker *(r* = 0.89)*.* We also show that although E_2_ and progesterone levels determined by the Gen III assays were lower than with the Gen II assays, the differences are only of potential clinical relevance for progesterone.

The lower progesterone levels measured with the Elecsys^®^ Progesterone Gen III assay versus the Elecsys^®^ Progesterone Gen II assay were particularly apparent in the lower concentration range (0–1 ng/mL). The magnitude of this difference was ~ 28% on day of ovulation triggering, which may have clinical implications. This may be particularly relevant if a “freeze-all” strategy is considered alongside segmentation of treatment [14], based on late follicular phase progesterone elevation to avoid a detrimental effect on pregnancy rates [15]. To further investigate the potential differences between the Elecsys^®^ Gen III and Gen II Progesterone assays, we compared measurements obtained using the assays and LC–MS/MS on the day of ovulation triggering. These data confirmed that Gen III results correlate better with LC–MS/MS than Gen II (Pearson’s *r* = 0.98 vs. 0.90, respectively) and show improved accuracy; this is likely due to the higher specificity and reduced cross-reactivity of antibodies used in the Gen III assay (Roche, data on file).

Previous findings support the use of a threshold of 1.5 ng/mL to define progesterone levels associated with pregnancy rates [1, 12, 13]; however, this threshold was obtained using the AxSYM system, and is not applicable to all assays or LC–MS/MS methods. Based on our findings, and if the threshold was applicable to the LC–MS/MS method used in the present study, the difference between progesterone results determined with each assay generation would translate into fewer misclassifications for Gen III versus Gen II. Clinicians changing from Gen II to Gen III progesterone assays should be aware of these differences when making clinical decisions, especially when measuring progesterone on the day of ovulation triggering. Our findings are generally consistent with earlier studies, where the Gen II assay demonstrated good correlation with LC–MS/MS (Pearson’s *r* = 0.987), but delivered higher values than LC–MS/MS at low concentrations [3]. Moreover, the Gen II assay showed good intra- and inter-assay precision, with coefficients of variation < 10% at all timepoints and concentrations tested, the lowest of all four analyzers assessed [3].

Good correlation was observed between the Elecsys^®^ Gen III and Gen II Estradiol assays. Although E_2_ levels were lower when measured with the Gen III assay compared with the Gen II assay above ~ 3000 pg/mL, the magnitude of this difference was not considered to be clinically relevant (~ 15% on day of ovulation triggering). This small difference is potentially explained by the use of additional samples in the standardization of the Gen III assay, which are better distributed to cover the measuring range (Roche, data on file). For the Gen III assay, E_2_ concentrations were similar for the poor-, normal-, and high-response groups when normalized to follicle count (e.g., 324–377 pg/mL for the GnRH antagonist patients); this was expected, as E_2_ production per follicle was expected to be the same across response groups. Taieb and colleagues previously found mean E_2_ concentrations per mature follicle of ~ 1200 pmol/L (327 pg/mL) for women undergoing GnRH agonism or antagonism in an assessment of another automated immunoassay [11]. Furthermore, in their retrospective review of 342 IVF cycles in patients undergoing a long GnRH agonist protocol in which the E_2_/follicle ratio was used, Mittal and colleagues found a positive correlation between the ratios of E_2_ to mature follicles (> 14 mm diameter) and E_2_ to oocytes (*p* = 0.0001), and showed that pregnancy rates increased when the E_2_ concentration per follicle was between 200 and 300 pg/mL [16].

Reliable assays that are specific to the measurement of E_2_ are required to precisely quantify E_2_ levels in women undergoing ovulation induction. These assays need to be able to measure both low baseline and high (i.e., 250–2000 pg/mL) E_2_ levels to assess treatment efficacy, the timing of ovulation triggering, and with cut-offs designed to abort cycles that risk hyperstimulation [8]. In addition, long-standing evidence strongly suggests that progesterone levels on the day of ovulation triggering are related to IVF outcomes [1, 2, 4, 17–23]. Some data have been contradictory [24–26], but the balance of evidence and the most recent systematic reviews [2, 27] support an association between progesterone levels on the day of final oocyte maturation and successful pregnancy outcomes. The Gen III Elecsys^®^ Estradiol and Progesterone assays will enable more accurate and reliable monitoring of E_2_ and progesterone levels to support clinical decision-making, including the adjustment of hormone levels during ovarian stimulation to optimize clinical outcome and the likelihood of pregnancy.

An important limitation of this study is that it was retrospective, and thus required reanalysis of frozen/thawed samples. Nonetheless, this is unlikely to have influenced our results given that previous findings support the use of frozen samples [28]. Furthermore, the intra- and inter-sonographer variation in the measurement of follicular diameter could have been associated with a potential source, although the use of experienced sonographers was expected to minimize this limitation. However, ultrasound follicular measurement was not at all associated with primary outcome assessment, which was the correlation between the two different generation assays.

Considering all this, our findings have important implications for clinical practice. During cycle monitoring, clinicians should be aware that progesterone levels on the day of final oocyte maturation are ~ 30% lower when measured with the Elecsys^®^ Gen III assay versus the Elecsys^®^ Gen II assay, probably due to the higher specificity and reduced cross-reactivity of antibodies used in the Gen III assay. Consequently, treatment strategies should be adapted accordingly taking into account this difference.

## Conclusions

The ESPRIT study expands the evidence surrounding E_2_ and progesterone testing in the IVF setting. The Elecsys^®^ Gen II and Gen III assays for the measurement of E_2_ demonstrated a high level of correlation; the correlation for progesterone measurement was slightly weaker. Progesterone levels measured by the Gen III assay were lower than those measured by the Gen II assay, and more similar to levels measured by LC–MS/MS. Clinicians changing from Elecsys^®^ Gen II to Gen III Elecsys^®^ assays for measuring progesterone should be aware of these differences, which are potentially clinically relevant, especially when measuring progesterone levels on day of ovulation triggering.

## Electronic supplementary material

Below is the link to the electronic supplementary material.

Supplementary file1 (PDF 421 kb)

Supplementary file2 (PDF 621 kb)

Supplementary file3 (PDF 669 kb)

Supplementary file4 (PDF 670 kb)

Supplementary file5 (PDF 443 kb)

Supplementary file6 (PDF 467 kb)

Supplementary file7 (PDF 471 kb)

Supplementary file8 (PDF 566 kb)

Supplementary file9 (PDF 622 kb)

## Data Availability

The data sets generated during and/or analysed during the current study are available from the corresponding author on reasonable request.
