# Supplementary material for: EStradiol and PRogesterone in In vitro ferTilization (ESPRIT): a multicenter study evaluating third- versus second-generation estradiol and progesterone immunoassays
Source: J Endocrinol Invest. 2020 Mar 13;43(9):1239–48. doi: 10.1007/s40618-020-01211-x (PMC7431432; doi:10.1007/s40618-020-01211-x)
Supplement: Supplementary file 1 — Supplementary file1 (PDF 421 kb) [file 40618_2020_1211_MOESM1_ESM.pdf]

EStradiol and PRogesterone in In vitro ferTilization (ESPRIT): a multicenter study evaluating third- versus second-generation estradiol and progesterone immunoassays

N.P. Polyzos • E. Anckaert • P. Drakopoulos • H. Tournaye • J. Schiettecatte • H. Donner • G. Bobba • G. Miles • W.D.J. Verhagen-Kamerbeek • E. Bosch

Corresponding author: Prof. Dr. Nikolaos P. Polyzos, Dexeus University Hospital, Gran Via Carles III, 71-75 - 08028 Barcelona, Spain. E-mail: nikpol@dexeus.com; n.polyzos@gmail.com

Journal of Endocrinological Investigation

Online resource 1: supplemental methods

Liquid chromatography–tandem-mass spectrometry (MS) testing

The calibrator/standard material for progesterone and the deuterated analog as internal standard, progesterone-d9, were obtained from Sigma Aldrich (Merck Group). Ultra liquid chromatography/MS grade solvents were from Biosolve B.V. (Valkenswaard, The Netherlands). Formic acid was purchased from Merck. Albumin/bovine plasma albumin-l and steroid-free serum (for eventual dilution needs of highly concentrated samples) were from Roche Diagnostics. The substance used as quality control, BCR-348R, was obtained from the Institute for Reference Materials and Measurements. Water was sourced from a Millipore Water Purifying System, and argon was delivered through the in-house gas supply system.

150 µL of serum and quality control samples were used in the analysis. Each sample was mixed thoroughly with 300 µL of internal standard/precipitation reagent for ~5 minutes using a sample mixer at room temperature. The samples were centrifuged for 5 minutes at 14,000 rpm at room temperature to separate precipitate debris from its supernatant. 400 µL of supernatant was transferred to centrifugation filters (Amicon Filters with a cutoff of 30 kDa), and again centrifuged for 50 minutes at 8000 rpm at room temperature. The filtrate was pipetted into high-performance liquid chromatography (HPLC) vials with micro inserts, and was injected in the HPLC–MS system. Calibrators were prepared similarly (but without the 5-minute centrifugation step), after production of a dilution series of standards covering the intended range of measurement (approximately 0, 15, and 150 ng/mL). After mixing calibrators with internal standard/precipitation reagent, the mixture was transferred to the centrifugation filters.

The chromatographic system consisted of a binary pump, column oven, and a temperature-regulated autosampler (Ultimate 3000 RSLC; Thermo Fisher Dionex). Analytes were separated from other components on an Agilent Zorbax Rx-C8 (2.1 × 150 mm, 5 µm) column. The mobile phases

were 50% acetonitrile/water + 0.1% formic acid (Eluent A) and 100% acetonitrile + formic acid (Eluent B). A linear gradient elution at a flow rate of 250 µL/min was performed with an increase from 30% Eluent B to 100% Eluent B within 9 minutes. The wash phase was performed for 3 minutes at 100% Eluent B and gradually, for 2.9 minutes, the Eluent proportions at the starting point were again attained. The total run time of the analysis was 15 minutes and the column temperature was maintained at 40°C. Injection volume was 50 µL.

The mass spectrometer used (TSQ Quantum Ultra; Thermo Fisher Scientific) was a triple quadrupole with a heated electrospray ionization probe, optimally tuned to detect the progesterone parent ion of m/z 315.15, the product ions of m/z 108.99 and m/z 96.99, the deuterated analog progesterone-d9 parent ion of m/z 324.20, and the product ions thereof of m/z 112.80 and m/z 99.80. Acquisition was in the positive selected reaction monitoring mode with centroid data collection in one scan event. The quantification software used was XCalibur Quan Browser (Thermo Fisher Scientific).
