# Supplementary material for: EStradiol and PRogesterone in In vitro ferTilization (ESPRIT): a multicenter study evaluating third- versus second-generation estradiol and progesterone immunoassays
Source: J Endocrinol Invest. 2020 Mar 13;43(9):1239–48. doi: 10.1007/s40618-020-01211-x (PMC7431432; doi:10.1007/s40618-020-01211-x)
Supplement: Supplementary file 2 — Supplementary file2 (PDF 621 kb) [file 40618_2020_1211_MOESM2_ESM.pdf]

# EStradiol and PRogesterone in In vitro ferTilization (ESPRIT): a multicenter study evaluating third- versus second-generation estradiol and progesterone immunoassays

N.P. Polyzos • E. Anckaert • P. Drakopoulos • H. Tournaye • J. Schiettecatte • H. Donner • G. Bobba • G. Miles • W.D.J. Verhagen-Kamerbeek • E. Bosch

**Corresponding author:** Prof. Dr. Nikolaos P. Polyzos, Dexeus University Hospital, Gran Via Carles III, 71-75 - 08028 Barcelona, Spain. E-mail: nikpol@dexeus.com; n.polyzos@gmail.com

Journal of Endocrinological Investigation

**Online resource 2: supplemental table 1** Baseline characteristics of patients (sites combined)

| Characteristic | Overall population (*N* = 230) | GnRH agonist protocol (*n* = 62) | GnRH antagonist protocol (*n* = 168) |
|---|---|---|---|
| Median age, years (range) | 36 (25–44) | 38 (26–42) | 36 (25–44) |
| Median age by response to ovarian stimulation, years (range) | | | |
| Poor (0–3 oocytes) | 39 (27–44) [*n* = 76] | 39 (27–42) [*n* = 32] | 39 (27–44) [*n* = 44] |
| Normal (4–15 oocytes) | 36 (25–44) [*n* = 94] | 36 (26–42) [*n* = 30] | 36 (25–44) [*n* = 64] |
| High (>15 oocytes) | 34 (25–43) [*n* = 60] | – | 34 (25–43) [*n* = 60] |
| Race, *n* (%) | | | |
| White/Caucasian | 156 (67.8) | 28 (45.2) | 128 (76.2) |
| Black/African American | 5 (2.2) | 4 (6.5) | 1 (0.6) |
| Asian | 2 (0.9) | 0 | 2 (1.2) |
| Other | 29 (12.6) | 14 (22.6) | 15 (8.9) |
| Not reported/unknown | 38 (16.5) | 16 (25.8) | 22 (13.1) |

*GnRH* gonadotropin-releasing hormone
