# Supplementary material for: EStradiol and PRogesterone in In vitro ferTilization (ESPRIT): a multicenter study evaluating third- versus second-generation estradiol and progesterone immunoassays
Source: J Endocrinol Invest. 2020 Mar 13;43(9):1239–48. doi: 10.1007/s40618-020-01211-x (PMC7431432; doi:10.1007/s40618-020-01211-x)
Supplement: Supplementary file 3 — Supplementary file3 (PDF 669 kb) [file 40618_2020_1211_MOESM3_ESM.pdf]

EStradiol and PRogesterone in In vitro ferTilization (ESPRIT): a multicenter study evaluating third- versus second-generation estradiol and progesterone immunoassays

N.P. Polyzos • E. Anckaert • P. Drakopoulos • H. Tournaye • J. Schiettecatte • H. Donner • G. Bobba • G. Miles • W.D.J. Verhagen-Kamerbeek • E. Bosch

Corresponding author: Prof. Dr. Nikolaos P. Polyzos, Dexeus University Hospital, Gran Via Carles III, 71-75 - 08028 Barcelona, Spain. E-mail: nikpol@dexeus.com; n.polyzos@gmail.com

Journal of Endocrinological Investigation

Online resource 3: supplemental table 2 Gen III follicle counts by GnRH treatment protocol (agonist/antagonist) and response to controlled ovarian stimulation group (poor/normal/high) for estradiol

			Follicle type												
**GnRH treatment protocol**	**Response to controlled ovarian stimulation group**	**Visit**	**Small (<12 mm)**			**Intermediate (12–15 mm)**			**Mature (≥16 mm)**						
			N	**Mean**	**Range**	***N***	**Mean**	**Range**	***N***	**Mean**	**Range**	***N*** [a]	**Mean**	**Median**	**Ratio** [b]
Agonist	Poor (0–3 oocytes)	1	0	NA	NA	0	NA	NA	0	NA	NA	16	16.6	15.4	NA
		2	32	3.4	0–8	32	1.6	0–4	0	NA	NA	32	301.3	183.3	NA
		3	29	2.2	0–6	29	1.7	0–5	29	1.6	0–4	29	670.9	649.7	NA
		Day of ovulation triggering	32	1.4	0–7	32	0.9	0–3	32	3.2	1–6	32	1086.2	899.2	337.47
	Normal (4–15 oocytes)	1	0	NA	NA	0	NA	NA	0	NA	NA	15	14.6	12.1	NA
		2	30	7.1	2–18	30	3.2	0–8	0	NA	NA	30	655.8	608.3	NA
		3	27	3.7	0–11	27	4.2	1–10	27	2.8	0–6	27	1425.9	1379.0	NA
		Day of ovulation triggering	30	2.1	0–10	30	3.0	0–8	30	5.5	3–12	30	2245.1	2176.0	410.69
Antagonist	Poor (0–3 oocytes)	1	14	4.2	1–9	0	NA	NA	0	NA	NA	42	61.6	41.2	NA
		2	44	3.7	1–11	44	1.5	0–6	0	NA	NA	44	471.4	416.6	NA
		3	30	1.9	0–8	30	2.4	0–6	30	1.2	0–7	30	806.4	672.5	NA
		Day of ovulation triggering	44	1.2	0–7	44	1.2	0–5	44	2.9	1–9	44	1087.0	943.2	376.60
	Normal (4–15 oocytes)	1	34	7.3	2–16	0	NA	NA	0	NA	NA	64	206.7	70.4	NA
		2	64	5.0	0–15	64	4.2	0–14	0	NA	NA	64	865.0	763.4	NA
		3	44	4.2	0–18	44	4.3	1–13	44	2.5	0–7	43	1396.1	1290.0	NA
		Day of ovulation triggering	64	2.0	0–12	64	3.1	0–12	64	6.2	1–13	59	2009.3	1792.0	323.91
	High (>15 oocytes)	1	30	11.3	0–22	0	NA	NA	0	NA	NA	59	385.3	144.0	NA
		2	60	10.6	0–37	60	7.6	0–19	0	NA	NA	60	1494.4	1329.0	NA
		3	40	8.4	0–37	40	11.5	3–22	40	3.5	0–15	36	2316.7	2220.0	NA
		Day of ovulation triggering	60	3.5	0–31	60	7.2	1–18	60	10.8	4–26	47	3516.6	2854.0	326.11

[a] Some individuals may have had fewer visits, causing differences in the counts. [b] Ratio = mean result on day of ovulation triggering/mean mature follicle count on day of ovulation triggering. *GnRH* gonadotropin-releasing hormone, *NA* not available
