# Supplementary material for: EStradiol and PRogesterone in In vitro ferTilization (ESPRIT): a multicenter study evaluating third- versus second-generation estradiol and progesterone immunoassays
Source: J Endocrinol Invest. 2020 Mar 13;43(9):1239–48. doi: 10.1007/s40618-020-01211-x (PMC7431432; doi:10.1007/s40618-020-01211-x)
Supplement: Supplementary file 4 — Supplementary file4 (PDF 670 kb) [file 40618_2020_1211_MOESM4_ESM.pdf]

EStradiol and PRogesterone in In vitro ferTilization (ESPRIT): a multicenter study evaluating third- versus second-generation estradiol and progesterone immunoassays

N.P. Polyzos • E. Anckaert • P. Drakopoulos • H. Tournaye • J. Schiettecatte • H. Donner • G. Bobba • G. Miles • W.D.J. Verhagen-Kamerbeek • E. Bosch

Corresponding author: Prof. Dr. Nikolaos P. Polyzos, Dexeus University Hospital, Gran Via Carles III, 71-75 - 08028 Barcelona, Spain. E-mail: nikpol@dexeus.com; n.polyzos@gmail.com

Journal of Endocrinological Investigation

Online resource 4: supplemental table 3 Gen III follicle counts by GnRH treatment protocol (agonist/antagonist) and response to controlled ovarian stimulation group (poor/normal/high) for progesterone

			Follicle type												
			Small (<12 mm)			Intermediate (12–15 mm)			Mature (≥16 mm)						
**GnRH treatment protocol**	**Response to controlled ovarian stimulation group**	**Visit**	***N***	**Mean**	**Range**	***N***	**Mean**	**Range**	***N***	**Mean**	**Range**	***N*[a]**	**Mean**	**Median**	**Ratio[b]**
Agonist	Poor (0–3 oocytes)	1	0	NA	NA	0	NA	NA	0	NA	NA	29	0.2	0.2	NA
		2	32	3.4	0–8	32	1.6	0–4	0	NA	NA	31	0.2	0.2	NA
		3	29	2.2	0–6	29	1.7	0–5	29	1.6	0–4	29	0.3	0.3	NA
		Day of ovulation triggering	32	1.4	0–7	32	0.9	0–3	32	3.2	1–6	32	0.5	0.5	0.16
	Normal (4–15 oocytes)	1	0	NA	NA	0	NA	NA	0	NA	NA	25	0.2	0.2	NA
		2	30	7.1	2–18	30	3.2	0–8	0	NA	NA	27	0.2	0.2	NA
		3	27	3.7	0–11	27	4.2	1–10	27	2.8	0–6	27	0.5	0.5	NA
		Day of ovulation triggering	30	2.1	0–10	30	3.0	0–8	30	5.5	3–12	30	0.7	0.7	0.14
Antagonist	Poor (0–3 oocytes)	1	14	4.2	1–9	0	NA	NA	0	NA	NA	40	0.3	0.3	NA
		2	44	3.7	1–11	44	1.5	0–6	0	NA	NA	39	0.2	0.2	NA
		3	30	1.9	0–8	30	2.4	0–6	30	1.2	0–7	29	0.4	0.4	NA
		Day of ovulation triggering	44	1.2	0–7	44	1.2	0–5	44	2.9	1–9	42	0.7	0.4	0.24
	Normal (4–15 oocytes)	1	34	7.3	2–16	0	NA	NA	0	NA	NA	58	0.2	0.2	NA
		2	64	5.0	0–15	64	4.2	0–14	0	NA	NA	61	0.3	0.3	NA
		3	44	4.2	0–18	44	4.3	1–13	44	2.5	0–7	42	0.5	0.5	NA
		Day of ovulation triggering	64	2.0	0–12	64	3.1	0–12	64	6.2	1–13	64	0.8	0.7	0.12
	High (>15 oocytes)	1	30	11.3	0–22	0	NA	NA	0	NA	NA	54	0.3	0.2	NA
		2	60	10.6	0–37	60	7.6	0–19	0	NA	NA	60	0.4	0.4	NA
		3	40	8.4	0–37	40	11.5	3–22	40	3.5	0–15	40	0.9	0.7	NA
		Day of ovulation triggering	60	3.5	0–31	60	7.2	1–18	60	10.8	4–26	60	1.1	1.0	0.11

[a]Some individuals may have had fewer visits, causing differences in the counts. [b]Ratio = mean result on day of ovulation triggering/mean mature follicle count on day of ovulation triggering*GnRH* gonadotropin-releasing hormone, *NA* not available
