# Supplementary material for: EStradiol and PRogesterone in In vitro ferTilization (ESPRIT): a multicenter study evaluating third- versus second-generation estradiol and progesterone immunoassays
Source: J Endocrinol Invest. 2020 Mar 13;43(9):1239–48. doi: 10.1007/s40618-020-01211-x (PMC7431432; doi:10.1007/s40618-020-01211-x)

EStradiol and PRogesterone in In vitro ferTilization (ESPRIT): a multicenter study evaluating third- versus second-generation estradiol and progesterone immunoassays

N.P. Polyzos • E. Anckaert • P. Drakopoulos • H. Tournaye • J. Schiettecatte • H. Donner • G. Bobba • G. Miles • W.D.J. Verhagen-Kamerbeek • E. Bosch

Corresponding author: Prof. Dr. Nikolaos P. Polyzos, Dexeus University Hospital, Gran Via Carles III, 71-75 - 08028 Barcelona, Spain. E-mail: nikpol@dexeus.com; n.polyzos@gmail.com

Journal of Endocrinological Investigation

Online resource 8: supplemental fig. 4 **a** Passing–Bablok regression analysis and **b** Bland–Altman plot for the comparison of progesterone concentrations measured using the Elecsys® Progesterone Gen II assay and LC-MS/MS. *2SD* two standard deviations, *LC-MS/MS* liquid chromatography–tandem-mass spectrometry

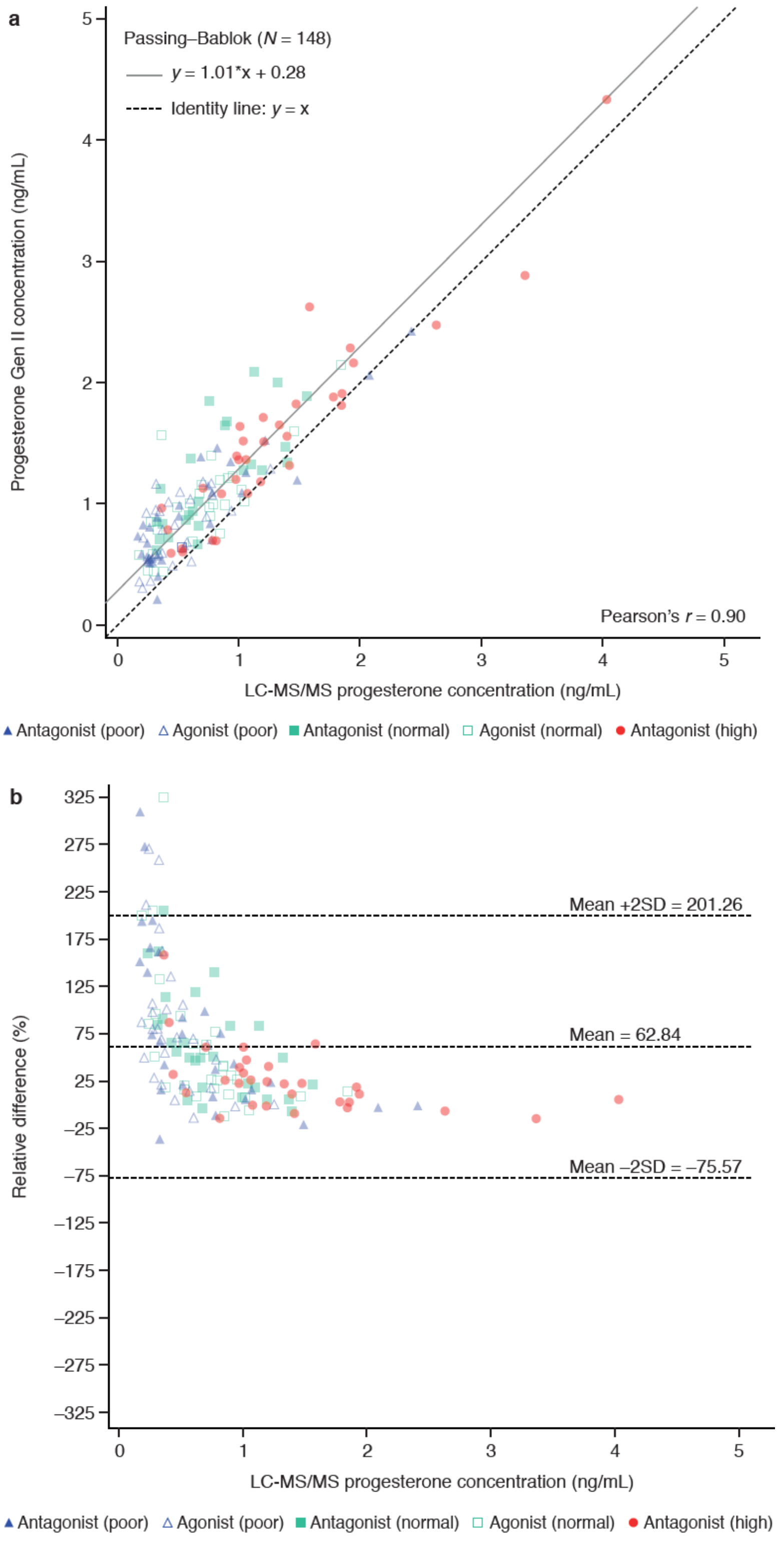

Supplement: Supplementary file 8 — Supplementary file8 (PDF 566 kb) [file 40618_2020_1211_MOESM8_ESM.pdf]
