# Supplementary material for: EStradiol and PRogesterone in In vitro ferTilization (ESPRIT): a multicenter study evaluating third- versus second-generation estradiol and progesterone immunoassays
Source: J Endocrinol Invest. 2020 Mar 13;43(9):1239–48. doi: 10.1007/s40618-020-01211-x (PMC7431432; doi:10.1007/s40618-020-01211-x)
Supplement: Supplementary file 9 — Supplementary file9 (PDF 622 kb) [file 40618_2020_1211_MOESM9_ESM.pdf]

EStradiol and PRogesterone in In vitro ferTilization (ESPRIT): a multicenter study evaluating third- versus second-generation estradiol and progesterone immunoassays

N.P. Polyzos • E. Anckaert • P. Drakopoulos • H. Tournaye • J. Schiettecatte • H. Donner • G. Bobba • G. Miles • W.D.J. Verhagen-Kamerbeek • E. Bosch

Corresponding author: Prof. Dr. Nikolaos P. Polyzos, Dexeus University Hospital, Gran Via Carles III, 71-75 - 08028 Barcelona, Spain. E-mail: nikpol@dexeus.com; n.polyzos@gmail.com

Journal of Endocrinological Investigation

Online resource 9: supplemental table 4 Relative difference in progesterone levels measured with the Elecsys® Gen III and Gen II assays compared with LC-MS/MS

			Relative difference			
**Comparison**	**Measurement range**	***N***	**Mean (SD)**	**Min, max**	**Lower limit (mean relative difference – 2SD)**	**Upper limit (mean relative difference + 2SD)**
Progesterone Gen III vs. LC-MS/MS (ng/mL)	Full	148	14.60 (23.79)	−47.19, 124.08	−32.99	62.18
	Truncated: 0–1	105	17.34 (26.68)	−47.19, 124.08	−36.03	70.70
	Truncated: >1–1.5	27	7.83 (14.18)	−13.88, 46.77	−20.52	36.19
	Truncated: >1.5	16	8.02 (9.09)	−4.41, 28.51	−10.15	26.20
Progesterone Gen II vs. LC-MS/MS (ng/mL)	Full	148	62.84 (69.21)	−34.69, 325.01	−75.57	201.26
	Truncated: 0–1	78	78.63 (73.99)	−34.69, 308.90	−69.35	226.62
	Truncated: >1–1.5	42	47.97 (55.04)	−19.49, 259.35	−62.12	158.05
	Truncated: >1.5	28	41.16 (65.65)	−14.02, 325.01	−90.15	172.47

Data are from samples from the UZ Brussel site on day of ovulation triggering
2SD two standard deviations, *LC-MS/MS* liquid chromatography–tandem-mass spectrometry,
SD standard deviation
